# Epigenetic therapy of acute myeloid leukemia using 5-aza-2'-deoxycytidine (decitabine) in combination with inhibitors of histone methylation and deacetylation

**DOI:** 10.1186/1868-7083-6-19

**Published:** 2014-10-01

**Authors:** Richard L Momparler, Sylvie Côté, Louise F Momparler, Youssef Idaghdour

**Affiliations:** 1 Département de Pharmacologie, Université de Montréal, 2900 Édouard-Montpetit, Montréal, QC H3T 1J4, Canada; 2 Centre de recherche, Service d’hématologie/oncologie, CHU-Saint-Justine, Montréal, Québec H3T 1C5, Canada; 3 Department of Biology, New York University, Saadiyat Island, PO Box 129188, Abu Dhabi, United Arab Emirates

**Keywords:** 5-aza-2’-deoxcytidine, 3-deazaneplanocin-A, Decitabine, Epigenetic therapy, EZH2, Histone deacetylase inhibitors, Myeloid leukemia

## Abstract

**Background:**

The silencing of tumor suppressor genes (TSGs) by aberrant DNA methylation occurs frequently in acute myeloid leukemia (AML). This epigenetic alteration can be reversed by 5-aza-2’-deoxcytidine (decitabine, 5-AZA-CdR). Although 5-AZA-CdR can induce complete remissions in patients with AML, most patients relapse. The effectiveness of this therapy may be limited by the inability of 5-AZA-CdR to reactivate all TSGs due to their silencing by other epigenetic mechanisms such as histone methylation or chromatin compaction. EZH2, a subunit of the polycomb repressive complex 2, catalyzes the methylation of histone H3 lysine 27 (H3K27) to H3K27me3. 3-Deazaneplanocin-A (DZNep), an inhibitor of methionine metabolism, can reactivate genes silenced by H3K27me3 by its inhibition of EZH2. In a previous report, we observed that 5-AZA-CdR, in combination with DZNep, shows synergistic antineoplastic action against AML cells. Gene silencing due to chromatin compaction is attributable to the action of histone deacetylases (HDAC). This mechanism of epigenetic gene silencing can be reversed by HDAC inhibitors such as trichostatin-A (TSA). Silent TSGs that cannot be reactivated by 5-AZA-CdR or DZNep have the potential to be reactivated by TSA. This provides a rationale for the use of HDAC inhibitors in combination with 5-AZA-CdR and DZNep to treat AML.

**Results:**

The triple combination of 5-AZA-CdR, DZNep, and TSA induced a remarkable synergistic antineoplastic effect against human AML cells as demonstrated by an *in vitro* colony assay. This triple combination also showed a potent synergistic activation of several key TSGs as determined by real-time PCR. The triple combination was more effective than the combination of two agents or a single agent. Microarray analysis showed that the triple combination generated remarkable changes in global gene expression.

**Conclusions:**

Our data suggest that it may be possible to design a very effective therapy for AML using agents that target the reversal of the following three epigenetic “lock” mechanisms that silence gene expression: DNA methylation, histone methylation, and histone deacetylation. This approach merits serious consideration for clinical investigation in patients with advanced AML.

## Background

Epigenetic mechanisms that control gene expression play an important role in leukemogenesis [1,2]. Aberrant DNA methylation that silences the expression of tumor suppressor genes (TSGs) occurs frequently in patients with acute myeloid leukemia (AML) and can be used to predict the outcome of therapy [3,4]. The importance of this epigenetic modification is illustrated by the use of the inhibitor of DNA methylation, 5-aza-2’-deoxcytidine (5-AZA-CdR, decitabine), to treat AML [5-8]. However, most AML patients induced into complete remission with 5-AZA-CdR will relapse, which provides a rationale to search for other agents to use in combination to increase the effectiveness of the therapy.

An attractive target in AML is the histone methyltransferase EZH2 [9], a subunit of the polycomb repressive complex 2 (PRC2). Repression of PRC2 target gene transcription occurs by the trimethylation of histone 3 at lysine 27 (H3K27) to H3K27me3 by EZH2 [10]. Overexpression of EZH2 is frequently observed in AML [11] and can block the differentiation of myeloid cells [12]. Additionally, the ectopic expression of EZH2 in murine hematopoietic cells results in excessive myeloid proliferation in bone marrow [13]. These findings suggest that EZH2 plays an important role in leukemogenesis. Reduction in the level of EZH2 by 3-deazaplanocin-A (DZNep), a competitive inhibitor of S-adenosyl-L-homocysteine hydrolase, inhibits the proliferation of AML cells [14]. There is a cross-talk between DNA and histone methylation [15] in which genes marked by the presence of EZH2 recruit DNMT1 [16] and show a higher frequency of DNA methylation in cancer [17]. These findings provided a rationale for using 5-AZA-CdR in combination with DZNep to treat AML by targeting two different epigenetic gene-silencing mechanisms. We previously reported that the combination of these epigenetic agents had potent antineoplastic interaction against AML cells [18].

In an attempt to optimize this epigenetic therapy of AML, we investigated whether the reversal of a third gene-silencing mechanism would further enhance the anti-leukemic action of 5-AZA-CdR plus DZNep. Epigenetic gene silencing can also be due to the conversion of open chromatin to a compact configuration by histone deacetylase (HDAC). HDAC inhibitors can reverse this block and show potential for the treatment of leukemia [19]. There is also a cross-talk between DNA methylation and HDAC to silence gene expression. The mechanism is due to the attachment of a 5-methylcytosine-binding protein to the target gene promoter, which is followed by the recruitment of HDAC [20]. The importance of this interaction in AML cells is shown by the synergistic activation of the TSG CDKN2B (p15) by 5-AZA-CdR plus the HDAC inhibitor, trichostatin-A (TSA) [21]. 5-AZA-CdR plus HDAC inhibitors show interesting antineoplastic activity against leukemia in both the laboratory [22] and clinic [23].

The cross-talk between DNA methylation, histone methylation, and histone deacetylation provides a rationale for using a combination of epigenetic agents that target these three gene-silencing mechanisms. In this report, we investigated the antineoplastic action of the combination of 5-AZA-CdR, DZNep, and TSA on AML cells. This combination of three epigenetic agents shows remarkable anti-leukemic activity against leukemic cells.

## Results

### Growth inhibition and reduction in survival of AML cells by combination of epigenetic agents

**Figure 1:**
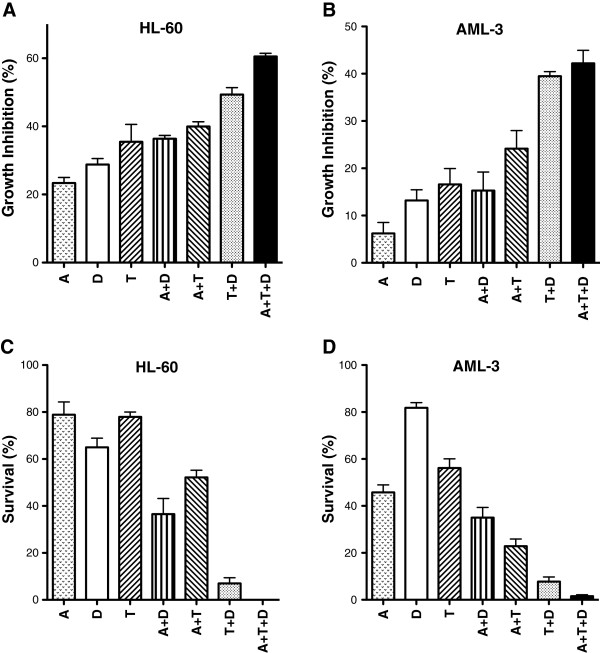
**Growth inhibition assay (A, B) and colony assay (C, D) of leukemic cells after sequential treatment with 5-AZA-CdR (A), DZNep (D), and/or TSA (T).** The leukemic cells were treated with 20 nM 5-AZA-CdR and, at 24 h, 500 nM (AML-3) or 1,000 nM (HL-60) DZNep and/or 40 nM (AML-3) or 80 nM (HL-60) nM TSA were added to the medium. At 48 h the cells were counted and placed in soft agar for colony assay to determine growth inhibition and cell survival, respectively. The results are expressed as mean ± SEM, n = 3. Statistical analysis for growth inhibition and reduction in survival: A + D + T > (A + D, A + T) *P* A<0.05 (one way ANOVA).

In previous reports from our laboratory we demonstrated that the antineoplastic activity of 5-AZA-CdR and DZNep or an HDAC inhibitor was synergistic on myeloid leukemia cell lines [18,22]. Our objective was to determine if the triple combination of these agents would be more effective than single or double combinations. The experimental approach was to treat AML cells with 5-AZA-CdR followed by the addition at 24 h of DZNep and TSA using concentrations as indicated in the legends. We used a sequential drug treatment that started with 5-AZA-CdR and was followed by histone modifiers because cell cycle analysis indicates that both DZNep and TSA can inhibit the progression of G1 cells into the S phase [14,19]. Because 5-AZA-CdR is S phase-specific, any block in the transit of the cells into the S phase induced by DZNep or TSA could allow some leukemic stem cells to escape the antineoplastic action of 5-AZA-CdR. At 48 h post drug treatment, the inhibition of cell growth was measured and the leukemic cells were plated in soft agar for colony assay. For both HL-60 (Figure 1A) and AML-3 (Figure 1B) cells, the triple combination produced a significantly greater inhibition of growth than either the single or double agents, except for the combination of DZNep plus TSA, which was not significant.A colony assay was performed to determine the survival of the leukemic cells produced by each individual agent and the different combinations of these agents. For both HL-60 (Figure 1C) and AML-3 (Figure 1D) cells, the triple combination produced a significantly greater reduction of survival than either the single or double agents. No HL-60 cells survived following treatment with the triple combination, whereas for AML-3 cells, only A<2% survival was observed. The combination of DZNep plus TSA showed remarkable anti-leukemic activity as indicated by only 7.0% and 7.8% survival for the HL-60 and AML-3 cells, respectively (Figure 1C and D). Single agent treatment alone showed >64% and >45% survival for HL-60 and AML-3 cells, respectively.

### Induction of apoptosis on AML cells by combination of epigenetic agents

**Figure 2:**
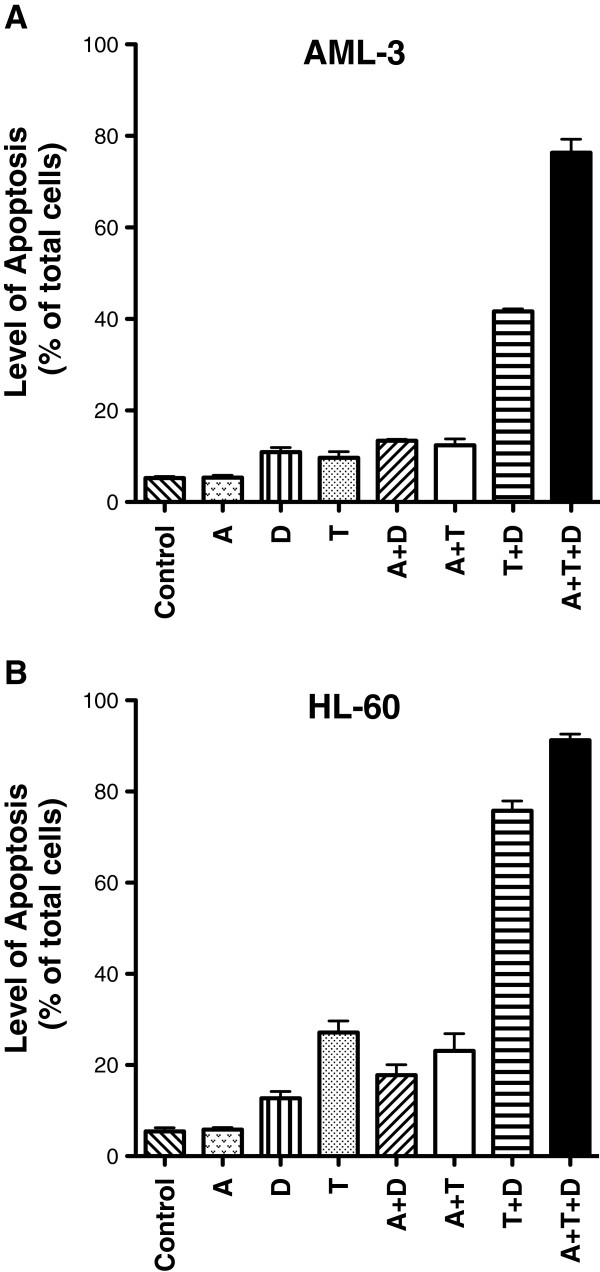
**Induction of apoptosis of leukemic cells after sequential treatment with 5-AZA-CdR (A), DZNep (D), and/or TSA (T).** AML-3 cells **(A)** and HL-60 cells **(B)** were treated with 20 nM 5-AZA-CdR and, at 24 h, 500 nM (AML-3) or 1,000 nM (HL-60) DZNep and/or 40 nM (AML-3) or 80 nM (HL-60) nM TSA were added to the medium. At 48 h the drugs were removed and at 72 h the cells were analyzed for induction of apoptosis using Annexin V staining. The results are expressed as mean ± SEM, n = 3. Statistical analysis for induction of apoptosis: AML-3 and HL-60 cells: A + D + T > (A + D, A + T, T + D) *P* A<0.05 (one way ANOVA).

Since drug resistance can be due to the suppression of apoptosis [24], we investigated the activity of the epigenetic agents alone and in combination on this parameter. DZNep was reported to induce apoptosis in myeloid leukemia cells [14] and tumor cells [25]. The induction of apoptosis by 5-AZA-CdR, DZNep, and TSA on the myeloid leukemia cell lines was evaluated by AnnexinV-PI staining (Figure 2). The concentration of these agents and exposure time were identical to that used for the growth and colony assay. For the AML-3 cells, as single agents or 5-AZA-CdR plus DZNep or plus TSA produced less than 15% apoptosis (Figure 2A). The combination of TSA plus DZNep produced 41.7% apoptosis as compared to 76.4% apoptosis by the triple combination, a synergistic interaction for both combinations as compared to the respective single agents or double combinations. The triple combination produced the most potent apoptotic activity. For the HL-60 cells, as single agents 5-AZA-CdR or DZNep produced less than 15%, and TSA alone produced 27.1% apoptosis (Figure 2B). 5-AZA-CdR plus DZNep or 5-AZA-CdR plus TSA produced 17.8% and 23.1% apoptosis, respectively. The TSA plus DZNep combination showed a synergistic induction of apoptosis of 75.8%, whereas the triple combination produced a greater apoptotic activity of 91.3%. For both these combinations the interaction was synergistic as compared to single agents or double combinations.

### Cell cycle perturbations of AML cells by combination of epigenetic agents

**Figure 3:**
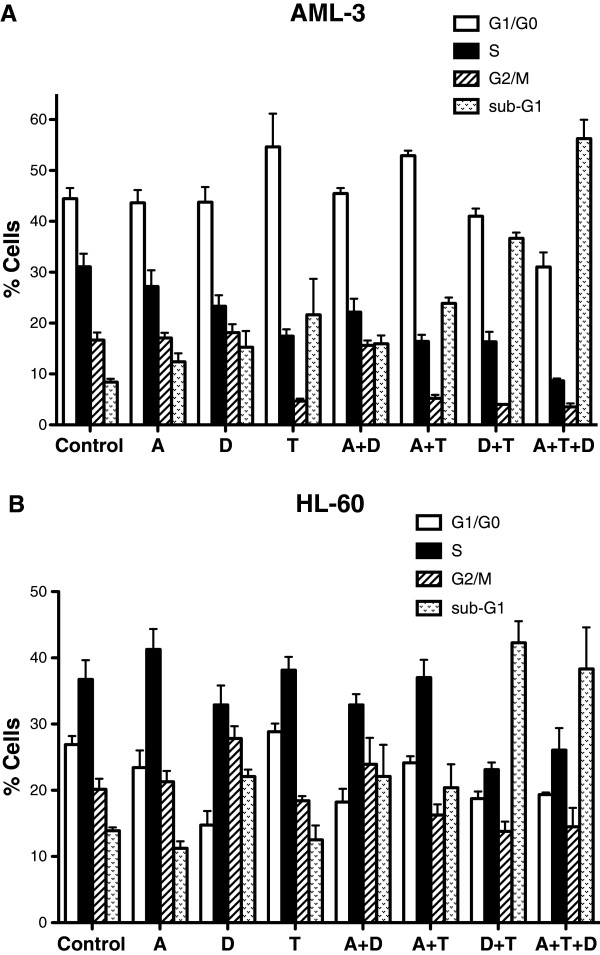
**Cell cycle analysis of leukemic cells after sequential treatment with 5-AZA-CdR (A), DZNep (D), and/or TSA (T).** AML-3 cells **(A)** and HL-60 cells **(B)** were treated with 20 nM 5-AZA-CdR and, at 24 h, 500 nM DZNep and/or 40 nM (AML-3) or 20 nM (HL-60) nM TSA were added to the medium. At 48 h cell cycle analysis was performed using flow cytometry of cells stained by propidium iodide. The results are expressed as mean ± SEM, n = 3.

Since both DZNep and HDAC inhibitors are known to inhibit cell cycle progression [14,19], we analyzed the effect of the epigenetic agents alone and in combination on the cell cycle of the HL-60 and AML-3 leukemic cells by flow cytometry (Figure 3). Drug concentrations were identical as in Figure 1 and analysis was performed at 48 h. For AML-3 cells, TSA alone increased the fraction of cells in G1/G0 to 55% as compared to 45% for the control and decreased the fraction of cells in the S phase to 18% as compared to the control of 32% (Figure 3A). These data suggest that TSA blocks the progression of G1 cells into the S phase and supports the rationale for sequential treatment of 5-AZA-CdR followed by TSA. For both cell lines, the double combination of DZNep plus TSA and the triple combination produced a remarkable synergistic increase in the fraction of cells in sub-G1 phase (Figure 3A and B). These latter data correlate with the induction of apoptosis by these combinations (Figure 2).

### Changes in gene expression in AML cells induced by combination of epigenetic agents

**Figure 4:**
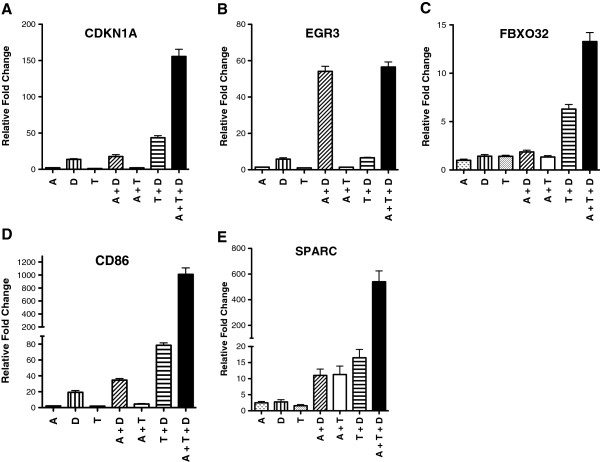
**Real-time quantitative PCR analysis of gene expression in HL-60 leukemic cells following sequential treatment with 5-AZA-CdR (A), DZNep (D), and/or TSA (T).** The cells were treated with 20 nM 5-AZA-CdR and, at 24 h, 1,000 nM DZNep and/or 80 nM TSA were added to the medium. At 48 h the drugs were removed and at 72 h RNA was isolated for RT-PCR analysis as described in Methods. The relative gene expression is shown as mean ± SEM, n = 3. Statistical analysis for increase in relative expression of: CDKN1A **(A)**, EGR3 **(B)**, FBXO32 **(C)**, CD86 **(D)**, and SPARC **(E)**: A + D + T > (A + D, A + T, T + D) *P* A<0.05 (one way ANOVA). EGR3 **(B)** A + D + T > A + D, n.s.

**Figure 5:**
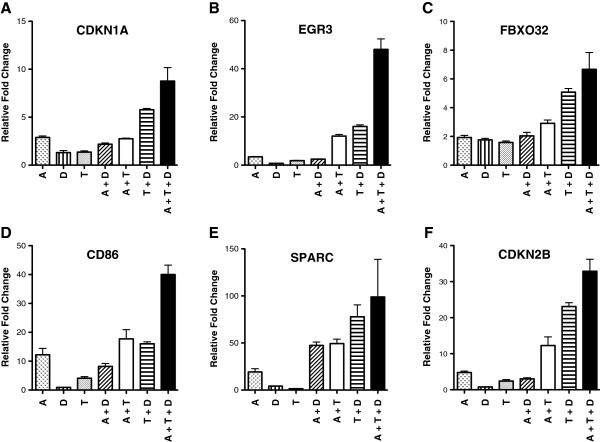
**Real-time quantitative PCR analysis of gene expression in AML-3 leukemic cells following sequential treatment with 5-AZA-CdR (A), DZNep (D), and/or TSA (T).** The cells were treated with 50 nM 5-AZA-CdR and, at 24 h, 1,000 nM DZNep and/or 40 nM TSA were added to the medium. At 48 h the drugs were removed and at 72 h RNA was isolated for RT-PCR analysis as described in Methods. The relative gene expression is shown as mean ± SEM, n = 3. Statistical analysis for increase in relative expression of: CDKN1A **(A)**, EGR3 **(B)**, FBXO32 **(C)**, CD86 **(D)**, and CDKN2B **(F)**: A + D + T > (A + D, A + T, T + D) *P* A<0.05 (one way ANOVA). For CDKN1A **(A)**, FBXO32 **(C)** and SPARC **(E)**: A + D + T > T + D, n.s.

In order to understand some of the molecular changes that take place in the leukemic cells, we analyzed the expression of several target genes that may play a role in leukemogenesis using real-time RT-PCR. For HL-60 cells, the triple combination produced a synergistic activation of the following genes: CDKN1A (p21), FBXO32, CD86, and SPARC (Figure 4). For AML-3 cells, the triple combination produced a synergistic activation of the following genes: CDKN1A (p21), EGR3, FBXO32, CD86, and CDKN2B (p15) (Figure 5). For both cell lines, each agent alone and in double combination produced an increase in expression of these genes that was much less than the triple combination.For the double combinations on HL-60 cells (Figure 4), the TSA plus DZNep combination showed a synergistic activation for CDKN1A, FBXO32, CD86, and SPARC as compared to each agent alone. The 5-AZA-CdR plus TSA combination showed a synergistic activation for SPARC. The 5-AZA-CdR plus DZNep combination showed a synergistic activation for EGR3, CD86, and SPARC. For the double combinations on AML-3 cells (Figure 5), the TSA plus DZNep combination showed a synergistic activation for all the genes. The 5-AZA-CdR plus TSA combination showed a synergistic activation for all the genes except CDKN1A. The 5-AZA-CdR plus DZNep combination showed a synergistic activation for only SPARC.

**Figure 6:**
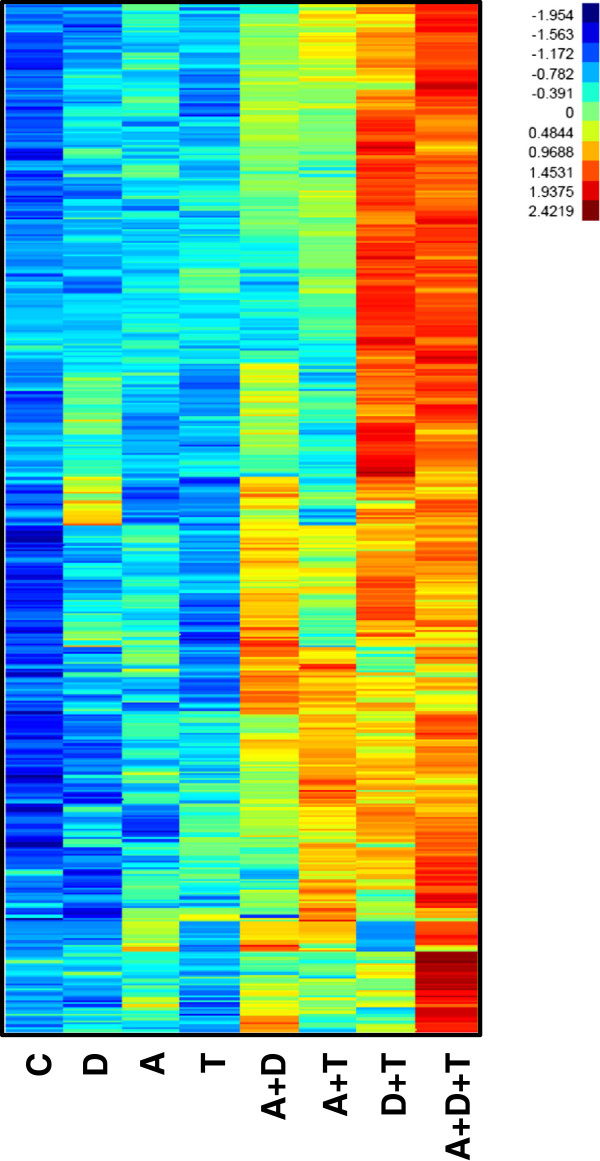
**Microarray analysis of gene expression in AML-3 leukemic cells after treatment with 5-AZA-CdR (A), DZNep (D), TSA (T), and combinations A + D, A + T, D + T, and A + D + T.** Control or untreated cells are labelled as **C**. The cells were treated with 50 nM 5-AZA-CdR, and, at 24 h, 1,000 nM DZNep and/or 40 nM TSA were added to the medium. At 48 h RNA was isolated for microarray analysis as described in Methods. The cluster diagram is a two-way clustering using the Ward method of the top 1,000 most up-regulated genes in response to the triple combination (A + D + T) relative to the control (C). The data plotted is log2 transformed, quantile normalized, and scaled to equal variance. The colors (blue to yellow to red) represent the spectrum of gene expression variation between conditions from low to high.

Microarray analysis of AML-3 cells following the treatment with the epigenetic agents was performed on genome-wide gene expression data and focused on the cohort of the top 1,000 genes that showed greatest increase in gene expression by the triple combination (Figure 6). Single agent and double agent treatment showed much less activation of gene expression as compared to triple combination of 5-AZA-CdR and DZNep plus TSA. The combination of DZNep plus TSA showed a remarkable increase in gene expression as compared to each single agent and to the other double combinations. The data on microarray are in accord with the results on survival, apoptosis, and gene expression analysis by real-time PCR (Figures 1, 2 and 5). (The complete microarray data are shown in Additional file 1: Table S1).

### Reduction in survival of AML cells by a combination of different epigenetic agents

**Figure 7:**
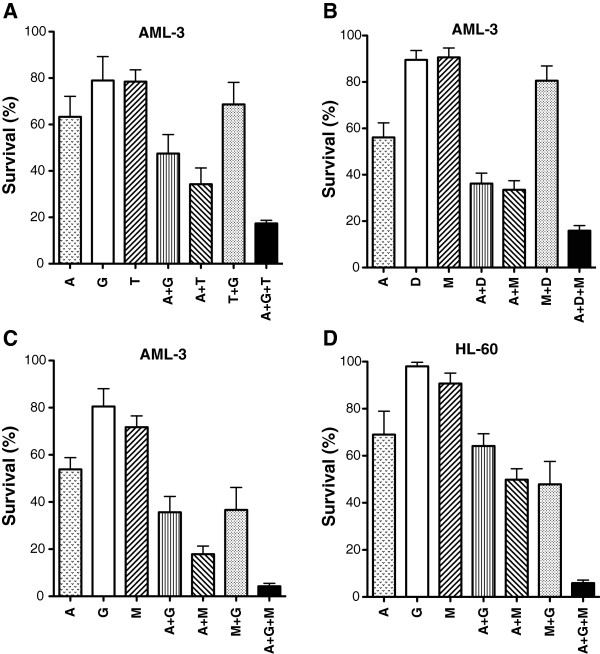
**Colony assay of AML-3 and HL-60 leukemic cells after sequential treatment with 5-AZA-CdR (A), GSK-126 (G) or DZNep (D), and/or TSA (T) or MS-275 (M).** The cells were treated with 20 nM 5-AZA-CdR and, at 24 h, the following drugs were added: **(A)** 1 μM GSK-126 and/or 20 nM TSA; **(B)** 500 nM DZNep and/or 200 nM MS-275; **(C)** 1 μM GSK-126 and/or 500 nM MS-275; **(D)** 5 μM GSK-126 and/or 1,000 nM MS-275. At 48 h the cells were counted and placed in soft agar for colony assay to determine cell survival. The results are expressed as mean ± SEM, n = 3. Statistical analysis for reduction in survival: **(A)** A + G + T > (T + G) *P* A<0.05; **(B)** A + D + M > (M + D) *P* A<0.05; **(C)** A + G + M > (A + G, M + G) *P* A<0.05; **(D)** A + G + M > (A + G, A + M, M + G) *P* A<0.05 (one way ANOVA).

DZNep is reported to be a global histone methyltransferase inhibitor that is not only specific for EZH2 [26]. In order to demonstrate that EZH2 is the primary target of DZNep, we replaced this analogue by GSK-126, a specific inhibitor of EZH2 [27]. For AML-3 cells, the combination of 5-AZA-CdR, GSK-126, and TSA showed a similar synergistic interaction in a colony assay as the triple combination that contained DZNep (Figure 7A). It should be noted that the DZNep plus TSA combination was much more potent than GSK-126 plus TSA. Replacement of TSA by the HDAC inhibitor MS-275 (entinostat) still showed a synergistic interaction for the triple combination on AML-3 cells in a colony assay (Figure 7B). The DZNep plus MS-275 was less potent than DZNep plus TSA. MS-275 is in clinical trial in patients with AML [28]. The results obtained with the triple combination of 5-Aza-CdR and GSK-126 plus MS-275 on AML-3 (Figure 7C) and HL-60 (Figure 7D) are similar to those in Figure 1.

## Discussion

There are several possible approaches that can be used to increase the therapeutic efficacy of AML therapy. The first approach is to optimize the dose schedule of 5-AZA-CdR [29,30]. Responses in patients with AML can be obtained with both low [5-8] and high doses of 5-AZA-CdR [31,32]. A second approach is to avoid the development of drug resistance to 5-AZA-CdR. We reported previously that drug resistance to this analogue due to deficiency in deoxycytidine kinase (the enzyme that activates this prodrug) could be overcome with the use of 3-deazauridine [33]. A third approach is to enhance the epigenetic action of 5-AZA-CdR on gene expression by its use in combination with other epigenetic agents.

In AML patients, gene-specific hypomethylation induced by 5-AZA-CdR does not always correlate with mRNA expression or leukemic blast count in bone marrow [34,35]. Genes silenced by DNA methylation may escape reactivation by 5-AZA-CdR if they contain the repressive marker, H3K27me3 [36]. If after 5-AZA-CdR treatment, the H3K27me3 mark is not removed, it can serve as a nidus for DNA re-methylation and gene re-silencing [36,37]. This removal of the H3K27me3 mark can be accomplished by the use of DZNep. The advantages of using DZNep in combination with 5-AZA-CdR are three fold. First, DZNep can reduce the H3K27me3 gene-silencing mark to activate the expression of genes that are demethylated, but not activated, by 5-AZA-CdR [36]. Second, DZNep can activate the expression of a unique cohort of genes compared to 5-AZA-CdR [38]. In both cases, a substantial number of genes are reactivated by the combination more so than either agent alone, resulting in greater anti-leukemic activity. Third, this combination of epigenetic agents can target the reactivation of genes that program cellular differentiation [12]. Our previous report shows that DZNep interacts synergistically with 5-AZA-CdR to activate gene expression and reduce leukemic cell survival [18]. This interesting drug interaction can be explained in part by the reversal of the “double lock” epigenetic mechanism for gene silencing by DNA and histone methylation.

Some investigators state that DZNep is not an ideal agent for targeted therapy because it is a global inhibitor of histone methyltransferases and is not specific for EZH2 [26]. However, it should be noted that DZNep shows potent antineoplastic activity against AML cells [14,18]. In support of EZH2 as a target for DZNep is our observation that replacement of this analogue by the specific inhibitor of EZH2, GSK-126 [27], provides similar results with respect to its interaction with 5-AZA-CdR on AML cells (Figure 7A). Overexpression of EZH2 in myeloid malignancies suggests that it functions as an oncogene [39]. However, loss-of-function mutations in EZH2 indicate that it may also function as a TSG in leukemia [39]. In the latter case, EZH2 inhibitors alone may not be appropriate agents for treating leukemia with this genetic abnormality. This question can be resolved by *in vitro* colony assays to test the sensitivity to DZNep of AML cells with EZH2 loss-of-function mutations. It should be noted that the action of 5-AZA-CdR and HDAC inhibitors may abolish the oncogenic potential of EZH2 inhibitors when used in combination. More studies are required to clarify the role of EZH2 mutations in the therapy of hematologic malignancies.

Another epigenetic mechanism of gene silencing is the conversion of open chromatin to a compact configuration by the action of HDAC. Its importance is illustrated by the positive interaction of 5-AZA-CdR with HDAC inhibitors to reactivate silent TSGs [21] and to inhibit the growth of leukemic cells [22]. Clinical trials on 5-AZA-CdR in combination with the HDAC inhibitor, valproic acid, was shown to induce complete response in some patients with AML [23,40]. There are also advantages to use HDAC inhibitors in combination with 5-AZA-CdR to treat AML. 5-AZA-CdR treatment only demethylates approximately half of the genes that are silenced by the presence of 5-methylcytosines in their promoter region [36,41]. This indicates that 5-AZA-CdR has a limited capacity to reactivate all silent TSGs and some leukemic stem cells escape its therapeutic action. It is important to note that HDAC inhibitors, as single agents in some cases, can activate genes silenced by DNA methylation [41]. Because the combination of HDAC inhibitors with 5-AZA-CdR has the potential to reactivate more silent TSGs than either agent alone, this will result in a marked enhancement of its anti-leukemic action. Our data are in accordance with this statement (Figure 1 and 7).

The triple combination of different epigenetic agents merits investigation in patients with advanced AML. This will require the approval of DZNep for clinical trials. TSA can be replaced by MS-275 (entinostat), an HDAC inhibitor that is approved for clinical studies and that shows some activity in patients with AML [28]. MS-275 had an interaction with 5-AZA-CdR that was similar to TSA with respect to the survival of leukemic cells (Figure 7B). Additionally, the combination of 5-AZA-CdR, GSK-126, and MS-275 also had a synergistic interaction against AML-3 (Figure 7C) and HL-60 cells (Figure 7D).

Curative therapy for AML requires the complete eradication of the proliferative potential of a very large number of leukemic stem cells. Leukemic cells containing TSGs silenced by more than one epigenetic mechanism may have the potential to escape 5-AZA-CdR therapy. The chemotherapeutic action of 5-AZA-CdR may be related not only to the reactivation of specific TSGs, but it may also be dependent on the total number of genes reactivated. This goal can be achieved by the use of a combination of agents that reverse the “triple lock” epigenetic mechanisms of gene silencing: DNA methylation, histone methylation, and deacetylation. It should be noted that each of these agents activates different cohorts of genes with minimal overlap [18,38]. We show that targeting the “triple lock” epigenetic silencing mechanisms by the combination of 5-AZA-CdR, DZNep, and TSA has a remarkable synergistic antineoplastic interaction on AML cells. The combination of these three epigenetic agents showed a synergistic reduction in the survival of AML cells as determined by a colony assay that was greater than observed with either single or double agent treatment (Figure 1). Additionally, the triple combination showed a remarkable synergistic induction of apoptosis in the AML cells (Figure 2). Resistance to the induction of apoptosis by chemotherapy is one of the hallmarks of cancer and can allow for the survival of malignant cells following drug treatment [24].

The notable changes in gene expression following the treatment of AML cells with the three epigenetic agents is most likely the major mechanism responsible for its chemotherapeutic action. Quantitative real-time PCR showed a remarkable synergistic reactivation by the triple combination of several genes: *CDKN1A* (*p21*), *EGR3*, *FBXO32*, *CD86*, *SPARC*, and *CDKN2B* (*p15*), which correlated with its antineoplastic action (Figures 4 and 5). All of these genes have some relationship with leukemogenesis [14,42-47]. The cyclin-dependent kinase inhibitor, *CDKN2B* (*p15*), is frequently silenced by DNA methylation in AML [42]. In addition, some AML cells with CDKN2B DNA methylation can also contain the H3K27me3 marker [43]. Microarray analysis shows that the triple combination activated the expression of a large set of genes to a much greater extent than either agent alone or the combinations of two agents (Figure 6). These data are similar to those obtained with breast carcinoma cells treated with the triple combination at higher concentrations and on a different schedule [38]. The remarkable changes in the global gene expression may play an important role in the anti-leukemic action of these epigenetic agents.

## Conclusions

In conclusion, the reversal of the "triple lock" mechanism of epigenetic gene silencing with specific agents that target DNA methylation, histone methylation, and deacetylation holds great promise for the treatment of AML. This combination therapy can reverse the key epigenetic aberrations that take place during leukemogenesis, and it has the capacity to eradicate the proliferative potential of leukemic stem cells. Additional research will clarify the importance of this interesting epigenetic therapy and define its future role in the therapy of AML.

## Methods

### Cell lines and materials

HL-60 and OCI-AML-3 human myeloid leukemic cells were obtained from ATCC and Dr Mark Minden, Ontario Cancer Institute, Toronto, respectively. The HL-60 and AML-3 cells were maintained in RPMI-1640-HEPES media and alpha-MEM (GIBCO), respectively. Fetal bovine serum (Wisent) was added to these media at a final concentration of 10%. 5-AZA-CdR was obtained from Dr Alois Piskala, Institute of Organic Chemistry, Czechoslovak Academy of Sciences, Prague. DZNep was kindly provided by Dr Victor E. Marquez, Chemical Biology Laboratory, National Cancer Institute, Frederick, MD, USA. 5-AZA-CdR and DZNep were dissolved in sterile phosphate buffer saline (PBS) pH 6.8 solution. TSA was acquired from Sigma and dissolved in ethanol. GSK-126 was obtained from Xcess Biosciences Inc. and dissolved in DMSO. MS-275 (entinostat) was provided by Schering (Germany) and dissolved in ethanol.

### Growth inhibition and colony assay

The HL-60 and AML-3 cells were treated with the indicated concentrations of drugs. Following the drug treatment, a cell count was performed using the Beckmann Model Z Coulter Counter. For colony assay, the cells were placed in 0.3% soft agar medium containing 20% serum. The number of colonies (>500 cells) was counted after 14 and 21 days of incubation. The cloning efficiency was in the range of 60 to 70%.

### Apoptosis analysis

Annexin V and propidium iodide (PI) staining were used to assess apoptosis and was determined using flow cytometry. The cells were treated as indicated. Twenty-four hours after the end of drug treatment, the cells were washed twice with cold PBS and resuspended in 1X Annexin V binding buffer (BD Biosciences Pharmingen). Then, 2 × 10^5^ cells were mixed gently with Annexin V-FITC (BD Biosciences Pharmingen) and PI solution (Sigma-Aldrich), and incubated for 15 min in the dark at room temperature. The cells were suspended in 1X Annexin V binding buffer and staining was immediately quantified using a BD LSR Fortessa flow cytometer (San Jose, CA, USA), and analyzed with the BD DIVA (San Jose, CA, USA) software program. A minimum of 10,000 cells within the gated region was analyzed per measurement.

### Cell cycle analysis

The cells were treated as indicated. After 48 h treatment, 4 × 10^6^ cells were washed twice with cold PBS containing 1% FBS. After ethanol fixation (at least 12 h at 4°C), cells were washed twice with cold PBS. The pelleted cells were stained by adding PBS containing PI (Sigma-Aldrich) and RNase A (Amersham). Staining was achieved in the dark at 4°C for 3 h prior to flow cytometry analysis using a BD LSR Fortessa flow cytometer, and analyzed with the Tree Star FlowJo (Ashland, OR, USA) software program to model the cell cycle assays. Fluorescence data were collected using a 561 nm laser excitation, and emission was collected using a 610/20 filter. Fluorescence data were obtained from at least 10,000 viable cells per sample.

### Analysis of gene expression

For quantitative PCR analysis, total RNA was reversed transcribed using the High Capacity cDNA Reverse Transcription Kit with random primers as described by the manufacturer (Applied Biosystems). The reaction mixture contained cDNA, specific primers for the target genes, Sybr green and TaqMan Fast Universal PCR Master Mix (Applied Biosystems). The ABI PRISM 7900HT Sequence Detection System (Applied Biosystems) was used to detect the amplification level. All reactions were run in triplicate and the average Cts were used for quantitation. The endogenous control was the *TATA-binding protein* (*TBP*) gene. The relative quantification of the target genes was determined using the ∆∆CT method. (The primers used for PCR analysis are shown in Additional file 2: Table S2).

For microarray analysis of gene expression, at 24 h after the end of drug treatment, total RNA was isolated from AML-3 cells using the RNAeasy Mini kit (Qiagen). Reverse transcription was performed using the Total Prep RNA Amplification kit (Ambion). The cDNA synthesis and *in vitro* transcription amplification were followed by microarray hybridization using the Human HT-12 v.4.0 Expression BeadChip kit following the manufacturer recommended protocols (Illumina). Three samples were replicated and all clustered adjacent to one another and the expression intensities were averaged in the statistical analysis. The raw intensities were extracted with the gene expression module in Illumina’s BeadStudio software. Expression intensities were log2 transformed and quantile normalized. A total of 30,442 with expression above background levels were retained for further analyses. Statistical analysis was performed using SAS 9.3 and JMP Genomics 6.1. Clustering analysis was done using the Ward method.

## Abbreviations

AML: Acute myeloid leukemia; 5-AZA-CdR: 5-aza-2’-deoxycytidine; DZNep: 3-deazaneplanocin-A; TSA: Trichostatin-A; TSG: Tumor suppressor gene.

## Competing interests

The authors declare that they have no competing interests.

## Authors’ contributions

RLM and SC designed the research. SC and LFM performed the experimental work. RLM and SC collected, analyzed, interpreted the data, and wrote the manuscript. YI analyzed and interpreted the microarray data. The manuscript was approved by all the co-authors.

## Supplementary Material

Additional file 1: Table S1 Excel file of gene expression by microarray analysis. Click here for file

Additional file 2: Table S2 Sequence of primers used for real-time PCR. Click here for file

## References

[B1] Esteller M Epigenetics in cancer N Engl J Med 2008 358 1148 1159 10.1056/NEJMra072067 18337604

[B2] Baylin SB Jones PA A decade of exploring the cancer epigenome-biological and translational implications Nat Rev Cancer 2011 11 726 734 10.1038/nrc3130 21941284 PMC3307543

[B3] Figueroa ME Lugthart S Li Y Erpelinck-Verschueren C Deng X Christos PJ Schifano E Booth J van Putten W Skrabanek L Campagne F Mazumdar M Greally JM Valk PJ Löwenberg B Delwel R Melnick A DNA methylation signatures identify biologically distinct subtypes in acute myeloid leukemia Cancer Cell 2010 17 13 27 10.1016/j.ccr.2009.11.020 20060365 PMC3008568

[B4] Deneberg S Grövdal M Karimi M Jansson M Nahi H Corbacioglu A Gaidzik V Döhner K Paul C Ekström TJ Hellström-Lindberg E Lehmann S Gene-specific and global methylation patterns predict outcome in patients with acute myeloid leukemia Leukemia 2010 24 932 941 10.1038/leu.2010.41 20237504

[B5] Issa JP Garcia-Manero G Giles FJ Mannari R Thomas D Faderl S Bayar E Lyons J Rosenfeld CS Cortes J Kantarjian HM Phase 1 study of low-dose prolonged exposure schedules of the hypomethylating agent 5-aza-2'- deoxycytidine (decitabine) in hematopoietic malignancies Blood 2004 103 1635 1640 10.1182/blood-2003-03-0687 14604977

[B6] Cashen AF Schiller GJ O'Donnell MR DiPersio JF Multicenter, phase II study of decitabine for the first-line treatment of older patients with acute myeloid leukemia J Clin Oncol 2010 28 556 561 10.1200/JCO.2009.23.9178 20026803

[B7] Blum W Garzon R Klisovic RB Schwind S Walker A Geyer S Liu S Havelange V Becker H Schaaf L Mickle J Devine H Kefauver C Devine SM Chan KK Heerema NA Bloomfield CD Grever MR Byrd JC Villalona-Calero M Croce CM Marcucci G Clinical response and miR-29b predictive significance in older AML patients treated with a 10-day schedule of decitabine Proc Natl Acad Sci U S A 2010 107 7473 7478 10.1073/pnas.1002650107 20368434 PMC2867720

[B8] Lübbert M Rüter BH Claus R Schmoor C Schmid M Germing U Kuendgen A Rethwisch V Ganser A Platzbecker U Galm O Brugger W Heil G Hackanson B Deschler B Döhner K Hagemeijer A Wijermans PW Döhner H A multicenter phase II trial of decitabine as first-line treatment for older patients with acute myeloid leukemia judged unfit for induction chemotherapy Haematologica 2012 97 393 401 10.3324/haematol.2011.048231 22058219 PMC3291594

[B9] Melnick A Epigenetic therapy leaps ahead with specific targeting of EZH2 Cancer Cell 2012 22 569 570 10.1016/j.ccr.2012.10.016 23153531 PMC3732786

[B10] Sauvageau M Sauvageau G Polycomb group proteins: Multi-faceted regulators of somatic stem cells and cancer Cell Stem Cell 2010 7 299 313 10.1016/j.stem.2010.08.002 20804967 PMC4959883

[B11] Xu F Li X Wu L Zhang Q Yang R Yang Y Zhang Z He Q Chang C Overexpression of the EZH2, RING1 and BMI1 genes is common in myelodysplastic syndromes: relation to adverse epigenetic alteration and poor prognostic scoring Ann Hematol 2011 90 643 653 10.1007/s00277-010-1128-5 21125401

[B12] Tanaka S Miyagi S Sashida G Chiba T Yuan J Mochizuki-Kashio M Suzuki Y Sugano S Nakaseko C Yokote K Koseki H Iwama A Ezh2 augments leukemogenicity by reinforcing differentiation blockage in acute myeloid leukemia Blood 2012 120 1107 1117 10.1182/blood-2011-11-394932 22677129

[B13] Herrera-Merchan A Arranz L Ligos JM de Molina A Dominguez O Gonzalez S Ectopic expression of the histone methyltransferase Ezh2 in haematopoietic stem cells causes myeloproliferative disease Nat Commun 2012 3 623 22233633 10.1038/ncomms1623

[B14] Fiskus W Wang Y Sreekumar A Buckley KM Shi H Jillella A Ustun C Rao R Fernandez P Chen J Balusu R Koul S Atadja P Marquez VE Bhalla KN Combined epigenetic therapy with histone methyltransferase EZH2 inhibitor 3-deazaneplanocin A and the histone deacetylase inhibitor panobinostat against human AML cells Blood 2009 114 2733 2743 10.1182/blood-2009-03-213496 19638619 PMC2756128

[B15] Viré E Brenner C Deplus R Blanchon L Fraga M Didelot C Morey L Van Eynde A Bernard D Vanderwinden JM Bollen M Esteller M Di Croce L de Launoit Y Fuks F The polycomb group protein EZH2 directly controls DNA methylation Nature 2006 439 871 874 16357870 10.1038/nature04431

[B16] Schlesinger Y Straussman R Keshet I Farkash S Hecht M Zimmerman J Eden E Yakhini Z Ben-Shushan E Reubinoff BE Bergman Y Simon I Cedar H Polycomb-mediated methylation on Lys27 of histone H3 pre-marks genes for de novo methylation in cancer Nat Genet 2007 39 232 236 10.1038/ng1950 17200670

[B17] Widschwendter M Fiegl H Egle D Mueller-Holzner E Spizzo G Marth C Weisenberger DJ Campan M Young J Jacobs I Laird PW Epigenetic stem cell signature in cancer Nature Genet 2007 39 157 158 10.1038/ng1941 17200673

[B18] Momparler RL Idaghdour Y Marquez VE Momparler LF Synergistic antileukemic action of inhibitors of DNA methylation and histone methylation Leukemia Res 2012 36 1049 1054 10.1016/j.leukres.2012.03.001 22472464

[B19] Schrump DS Cytotoxicity mediated by histone deacetylases inhibitors in cancer cells: Mechanisms and potential clinical implications Clin Cancer Res 2009 15 3947 3957 10.1158/1078-0432.CCR-08-2787 19509170 PMC6354580

[B20] Jones PL Veenstra GJ Wade PA Vermaak D Kass SU Landsberger N Strouboulis J Wolffe AP Methylated DNA and MeCP2 recruit histone deacetylase to repress transcription Nat Genet 1998 19 187 191 10.1038/561 9620779

[B21] Cameron EE Bachman KE Myöhänen S Herman JG Baylin SB Synergy of demethylation and histone deacetylase inhibition in the re-expression of genes silenced in cancer Nat Genet 1999 21 103 107 10.1038/5047 9916800

[B22] Lemaire M Momparler LF Farinha NJ Bernstein M Momparler RL Enhancement of antineoplastic action of 5-aza-2’-deoxycytidine by phenylbutyrate on L1210 leukemic cells Leukemia Lymphoma 2004 45 147 154 10.1080/1042819031000149304 15061212

[B23] Garcia-Manero G Kantarjian HM Sanchez-Gonzalez B Yang H Rosner G Verstovsek S Rytting M Wierda WG Ravandi F Koller C Xiao L Faderl S Estrov Z Cortes J O’brien S Estey E Bueso-Ramos C Fiorentino J Jabbour E Issa JP Phase 1/2 study of the combination of 5-aza-2'-deoxycytidine with valproic acid in patients with leukemia Blood 2006 108 3271 3279 10.1182/blood-2006-03-009142 16882711 PMC1895437

[B24] Hanahan D Weinberg RL Hallmarks of cancer: The next generation Cell 2012 44 647 664 10.1016/j.cell.2011.02.013 21376230

[B25] Tan J Yang X Zhuang L Jiang X Chen W Lee PL Karuturi RK Tan PB Liu ET Yu Q Pharmacologic disruption of polycomb-repressive complex 2-mediated gene repression selectively induces apoptosis in cancer cells Gene Develop 2007 21 1050 1063 10.1101/gad.1524107 17437993 PMC1855231

[B26] Miranda TB Cortez CC Yoo CB Liang G Abe M Kelly TK Marquez VE Jones PA DZNep is a global histone methylation inhibitor that reactivates developmental genes not silenced by DNA methylation Mol Cancer 2009 8 1579 1588 10.1158/1535-7163.MCT-09-0013 PMC3186068 19509260

[B27] McCabe MT Ott HM Ganji G Korenchuk S Thompson C Van Aller GS Liu Y Graves AP Della Pietra A III Diaz E LaFrance LV Mellinger M Duquenne C Tian X Kruger RG McHugh CF Brandt M Miller WH Dhanak D Verma SK Tummino PJ Creasy CL EZH2 inhibition as a therapeutic strategy for lymphoma with EZH2-activating mutations Nature 2012 492 108 112 10.1038/nature11606 23051747

[B28] Gojo I Jiemjit A Trepel JB Sparreboom A Figg WD Rollins S Tidwell ML Greer J Chung EJ Lee MJ Gore SD Sausville EA Zwiebel J Karp JE Phase 1 and pharmacologic study of MS-275, a histone deacetylase inhibitor, in adults with refractory and relapsed acute leukemias Blood 2007 109 2781 2790 17179232 10.1182/blood-2006-05-021873 PMC1852211

[B29] Momparler RL Côté S Eliopoulos N Pharmacological approach for optimization of the dose-schedule of 5-aza-2’-deoxycytidine (Decitabine) for the therapy of leukemia Leukemia 1997 11 175 180 10.1038/sj.leu.2400550 9009076

[B30] Lemaire M Chabot GG Raynal NJ Momparler LF Hurtubise A Bernstein ML Momparler RL Importance of dose-schedule of 5-aza-2'-deoxycytidine for the epigenetic therapy of cancer BMC Cancer 2008 8 128 10.1186/1471-2407-8-128 18454857 PMC2386792

[B31] Momparler RL Rivard GE Gyger M Clinical trial on 5-aza-2-deoxycytidine in patients with acute leukemia Pharmac Ther 1986 30 277 286 10.1016/0163-7258(85)90052-x 2433702

[B32] Richel DJ Colly LP Kluin-Nelemans JC Willemze R The antileukaemic activity of 5-aza-2 deoxycytidine (Aza-dC) in patients with relapsed and resistant leukaemia Br J Cancer 1991 64 144 148 10.1038/bjc.1991.258 1713050 PMC1977302

[B33] Raynal NJ Momparler LF Rivard GE Momparler RL 3-Deazauridine enhances the antileukemic action of 5-aza-2'-deoxycytidine and targets drug-resistance due to deficiency in deoxycytidine kinase Leuk Res 2010 35 110 118 20510451 10.1016/j.leukres.2010.04.014

[B34] Claus R Pfeifer D Almstedt M Zucknick M Hackanson B Plass C Lübbert M Decitabine induces very early in vivo DNA methylation changes in blasts from patients with acute myeloid leukemia Leuk Res 2013 37 190 196 10.1016/j.leukres.2012.10.015 23158571

[B35] Yan P Frankhouser D Murphy M Tam HH Rodriguez B Curfman J Trimarchi M Geyer S Wu YZ Whitman SP Metzeler K Walker A Klisovic R Jacob S Grever MR Byrd JC Bloomfield CD Garzon R Blum W Caligiuri MA Bundschuh R Marcucci G Genome-wide methylation profiling in decitabine-treated patients with acute myeloid leukemia Blood 2012 120 2466 2474 10.1182/blood-2012-05-429175 22786882 PMC3448258

[B36] Si J Boumber YA Shu J Qin T Ahmed S He R Jelinek J Issa JP Chromatin remodeling is required for gene reactivation after decitabine-mediated DNA hypomethylation Cancer Res 2010 70 6968 6977 10.1158/0008-5472.CAN-09-4474 20713525 PMC2932851

[B37] McGarvey KM Fahrner JA Greene E Martens J Jenuwein T Baylin SB Silenced tumor suppressor genes reactivated by DNA demethylation do not return to a fully euchromatic chromatin state Cancer Res 2006 66 3541 3549 10.1158/0008-5472.CAN-05-2481 16585178

[B38] Sun F Chan E Wu Z Yang X Marquez VE Yu Q Combinatorial pharmacologic approaches target EZH2-mediated gene repression in breast cancer cells Mol Cancer Ther 2009 8 3191 3202 10.1158/1535-7163.MCT-09-0479 19934278 PMC2794891

[B39] Lund K Adams PD Copland M EZH2 in normal and malignant hematopoiesis Leukemia 2014 28 44 49 10.1038/leu.2013.288 24097338

[B40] Blum W Klisovic RB Hackanson B Liu Z Liu S Devine H Heerema NA Murgo A Chan KK Grever MR Byrd JC Marcucci G Phase I study of decitabine alone or in combination with valproic acid in acute myeloid leukemia J Clin Oncol 2007 25 3884 3891 10.1200/JCO.2006.09.4169 17679729

[B41] Raynal NJ Si J Taby RF Gharibyan V Ahmed S Jelinek J Estécio MR Issa JP DNA methylation does not stably lock gene expression but instead serves as a molecular mark for gene silencing memory Cancer Res 2012 72 1170 1181 10.1158/0008-5472.CAN-11-3248 22219169 PMC3294136

[B42] Agrawal S Unterberg M Koschmieder S Zur Stadt U Brunnberg U Verbeek W Büchner T Berdel WE Serve H Müller-Tidow C DNA methylation of tumor suppressor genes in clinical remission predicts the relapse risk in acute myeloid leukemia Cancer Res 2007 67 1370 1377 10.1158/0008-5472.CAN-06-1681 17283175

[B43] Paul TA Bies J Small D Wolff L Signatures of polycomb repression and reduced H3K4 trimethylation are associated with p15INK4b DNA methylation in AML Blood 2010 115 3098 3108 10.1182/blood-2009-07-233858 20190193 PMC2858468

[B44] Yasunaga J Taniguchi Y Nosaka K Yoshida M Satou Y Sakai T Mitsuya H Matsuoka M Identification of aberrantly methylated genes in association with adult T-cell leukemia Cancer Res 2004 64 6002 6009 10.1158/0008-5472.CAN-04-1422 15342380

[B45] Re F Arpinati M Testoni N Ricci P Terragna C Preda P Ruggeri D Senese B Chirumbolo G Martelli V Urbini B Baccarani M Tura S Rondelli D Expression of CD86 in acute myelogenous leukemia is a marker of dendritic/monocytic lineage Exp Hematol 2002 30 126 134 10.1016/S0301-472X(01)00768-8 11823047

[B46] Giallongo C La Cava P Tibullo D Barbagallo I Parrinello N Cupri A Stagno F Consoli C Chiarenza A Palumbo GA Di Raimondo F SPARC expression in CML is associated to imatinib treatment and to inhibition of leukemia cell proliferation BMC Cancer 2013 13 60 10.1186/1471-2407-13-60 23383963 PMC3570354

[B47] Gartel AL P21(WAF1/CIP1) may be a tumor suppressor after all Cancer Biol Ther 2007 6 1171 1172 17726367 10.4161/cbt.6.8.4712

