# Supplementary material for: Epigenetic therapy of acute myeloid leukemia using 5-aza-2'-deoxycytidine (decitabine) in combination with inhibitors of histone methylation and deacetylation
Source: Clin Epigenetics. 2014 Oct 1;6(1):19. doi: 10.1186/1868-7083-6-19 (PMC4194463; doi:10.1186/1868-7083-6-19)
Supplement: Additional file 2: Table S2 — Sequence of primers used for real-time PCR. [file 1868-7083-6-19-S2.doc]

**Table S2**: List of primers for real-time RT-PCR

| **Gene symbol** | **Reference Sequence** | **Forward primer** | **Reverse primer** |
| --- | --- | --- | --- |
| CDKN1A | NM_000389.3 | tcactgtcttgtacccttgtgc | ggcgtttggagtggtagaaa |
| EGR3 | NM_004430 | caatctgtaccccgaggaga | cagaccgatgtccattacattc |
| FBXO32 | NM_058229 | gcagcagctgaacaacattc | cacaaaggcaggtcagtgaa |
| CD86 | NM_175862 | ggcctcgcaactcttataaatg | ttttctcttttcttggtctgttcac |
| SPARC | NM_003118 | gcagaggaaaccgaagagg | cttgccgtgtttgcagtg |
| CDKN2B | NM_004936 | caacggagtcaaccgtttc | ggtgagagtggcagggtct |
| TBP | NM_003194.3 | gaacatcatggatcagaacaaca | atagggattccgggagtcat |
| HPRT | NM_000194.2 | tgatagatccattcctatgactgtaga | caagacattctttccagttaaagttg |
